# Plasma Exchange-Based Non-bioartificial Liver Support System Improves the Short-Term Outcomes of Patients With Hepatitis B Virus-Associated Acute-on-Chronic Liver Failure: A Multicenter Prospective Cohort Study

**DOI:** 10.3389/fmed.2021.779744

**Published:** 2021-11-16

**Authors:** Yuan-yuan Chen, Hai Li, Bao-yan Xu, Xin Zheng, Bei-ling Li, Xian-bo Wang, Yan Huang, Yan-hang Gao, Zhi-ping Qian, Feng Liu, Xiao-bo Lu, Jia Shang, Hai Li, Shao-yang Wang, Yin-hua Zhang, Zhong-ji Meng, Chinese Chronic Liver Failure (CLIF) Consortium, Shan Yin

**Affiliations:** ^1^Department of Infectious Diseases, Hubei Clinical Research Center for Precise Diagnosis and Therapy of Liver Cancer, Taihe Hospital, Hubei University of Medicine, Shiyan, China; ^2^Key Laboratory of Gastroenterology and Hepatology, Department of Gastroenterology, Renji Hospital, School of Medicine, Shanghai Institute of Digestive Disease, Shanghai Jiao Tong University, Chinese Ministry of Health (Shanghai Jiao Tong University), Shanghai, China; ^3^Department of Infectious Diseases, Southwest Hospital, Third Military Medical University (Army Medical University), Chongqing, China; ^4^Department of Infectious Diseases, Institute of Infection and Immunology, Union Hospital, Tongji Medical College, Huazhong University of Science and Technology, Wuhan, China; ^5^Hepatology Unit, Department of Infectious Diseases, Nanfang Hospital, Southern Medical University, Guangzhou, China; ^6^Center of Integrative Medicine, Beijing Ditan Hospital, Capital Medical University, Beijing, China; ^7^Hunan Key Laboratory of Viral Hepatitis, Department of Infectious Disease, Xiangya Hospital, Central South University, Changsha, China; ^8^Department of Hepatology, The First Hospital of Jilin University, Changchun, China; ^9^Department of Liver Intensive Care Unit, Shanghai Public Health Clinical Centre, Fudan University, Shanghai, China; ^10^Department of Infectious Diseases and Hepatology, The Second Hospital of Shandong University, Jinan, China; ^11^Liver Disease Center, First Affiliated Hospital of Xinjiang Medical University, Urumqi, China; ^12^Department of Infectious Diseases, Henan Provincial People's Hospital, Zhengzhou, China; ^13^Infectious Disease Center, Affiliated Hospital of Logistics University of People's Armed Police Force, Tianjin, China; ^14^Department of Infectious Diseases, Fuzhou General Hospital of Nanjing Military Command, Fuzhou, China

**Keywords:** acute-on-chronic liver failure, hepatitis B virus, plasma exchange, survival rate, propensity score matching

## Abstract

**Background and aims:** Hepatitis B virus-associated acute-on-chronic liver failure (HBV-ACLF) is a complicated syndrome with extremely high short-term mortality. Whether plasma exchange (PE) improves HBV-ACLF outcomes remains controversial. Here, PE-based non-bioartificial liver support system (NB-ALSS) effects on short-term HBV-ACLF patient outcomes were investigated.

**Materials and methods:** HBV-ACLF patients from Chinese Acute-on-chronic Liver Failure (CATCH-LIFE) cohort receiving standard medical therapy (SMT) alone or PE-based NB-ALSS in addition to SMT were allocated to SMT and SMT+PE groups, respectively; propensity score matching (PSM) was used to eliminate confounding bias. Short-term (28/90-day and 1-year) survival rates were calculated (Kaplan-Meier).

**Results:** In total, 524 patients with HBV-ACLF were enrolled in this study; 358 received SMT alone (SMT group), and the remaining 166 received PE-based NB-ALSS in addition to SMT (SMT+PE group). PSM generated 166 pairs of cases. In the SMT+PE group, 28-day, 90-day, and 1-year survival rates were 11.90, 8.00, and 10.90%, respectively, higher than those in the SMT group. Subgroup analysis revealed that PE-based NB-ALSS had the best efficacy in patients with ACLF grade 2 or MELD scores of 30–40 (MELD grade 3). In MELD grade 3 patients who received SMT+PE, 28-day, 90-day, and 1-year survival rates were improved by 18.60, 14.20, and 20.10%, respectively. According to multivariate Cox regression analysis, PE-based NB-ALSS was the only independent protective factor for HBV-ACLF patient prognosis at 28 days, 90 days, and 1 year (28 days, HR = 0.516, *p* = 0.001; 90 days, HR = 0.663, *p* = 0.010; 1 year, HR = 0.610, *p* = 0.051). For those who received SMT+PE therapy, PE-based NB-ALSS therapy frequency was the only independent protective factor for short-term prognosis (28-day, HR = 0.597, *p* = 0.001; 90-day, HR = 0.772, *p* = 0.018).

**Conclusions:** This multicenter prospective study showed that the addition of PE-based NB-ALSS to SMT improves short-term (28/90 days and 1-year) outcomes in patients with HBV-ACLF, especially in MELD grade 3 patients. Optimization of PE-based NB-ALSS may improve prognosis or even save lives among HBV-ACLF patients.

## Introduction

Acute-on-chronic liver failure (ACLF) is a clinical syndrome that occurs on the basis of chronic liver disease and is characterized by rapidly progressing organ failure and high short-term mortality (1). China is a high endemic area of hepatitis B virus (HBV). Indeed, there are 78 million HBV carriers and 28 million chronic hepatitis B (CHB) patients in China (2), and HBV-associated acute-on-chronic liver failure (HBV-ACLF) is the most common type of liver failure. A study by Xie et al. showed that 96.5% of ACLF patients in Southwest China were HBV-ACLF (3); in contrast, alcoholism and hepatitis C virus (HCV) infection are the most common causes of ACLF in Western developed countries (4, 5). The 28-day mortality is >15% in all ACLF patients and 40% in HBV-ACLF patients (6, 7).

Shi et al.'s study showed that HBV reactivation is the most common inducement of HBV-ACLF (8). The occurrence of drug resistance to nucleos(t)ide analogs (NAs) and NAs withdrawal without authorization are the main causes of HBV reactivation (9). Li et al. found that the immune metabolic disorder caused by HBV was the core axis of the occurrence and progression of HBV-ACLF (10). Bacterial infection is another common inducement of HBV-ACLF. Innate immune disorders and subsequent systemic inflammatory response syndrome (SIRS) not only drive the occurrence and progression of HBV-ACLF (11–13), but also lead to multiple organ failure in HBV-ACLF patients (14), and eventually damage the defense system of host immune cells and impair immune function, rendering HBV-ACLF patients more prone to secondary infection, which will further aggravate organ dysfunction and cause a sudden rise in the mortality of these patients (15–17).

Liver transplantation (LT) is currently the most effective therapy for ACLF. However, the shortage of donors and the high cost limit its clinical application (18, 19). Although NAs effectively inhibit HBV replication in CHB patients and reduce the 90-day mortality of HBV-ACLF patients, they are effective only in patients with a MELD score of 20–30 (20, 21). Various non-bioartificial liver support systems (NB-ALSSs) have since the late 1950's been utilized to treat liver failure, and plasma exchange (PE) is the most commonly applied mode of NB-ALSS in China. PE removes HBV-ACLF patient plasma rich in toxic metabolites and pro-inflammatory cytokines and compensates with the same volume of fresh frozen plasma supplemented with coagulation factors, albumin, immunoglobulin and other essential components to improve the liver microenvironment and to facilitate liver regeneration and functional recovery (22–24). A number of studies have shown that NB-ALSS, especially PE, is able to prolong the survival of patients with acute liver failure (ALF) or ACLF and improve their short-term outcomes (22, 25, 26). Case-control studies have shown that PE prolongs the survival of patients with ACLF and thus plays a role in bridging LT but that it does not improve short-term outcomes in patients with ACLF (27–29). There were also studies showing that PE improves the laboratory parameters and clinical symptoms of ACLF patients, without reporting whether PE improve outcomes (30, 31). Klementina et al. conducted a network meta-analysis and found that only PE therapy significantly improved 90-day outcomes in ACLF patients (32). Therefore, it remains controversial whether PE-based NB-ALSS therapy improves the outcomes of patients with HBV-ACLF.

Based on the two large prospective multicenter cohorts of the Chinese acute-on-chronic liver failure (CATCH-LIFE) study (33, 34), the present study focused on whether PE-based NB-ALSS therapy improves short-term (28/90 days and 1-year) outcomes of patients with HBV-ACLF and examined factors influencing the efficacy of PE-based NB-ALSS therapy for these patients.

## Materials and Methods

### Patients

The HBV-ACLF patients enrolled in this study were all from the CATCH-LIFE study. The CATCH-LIFE study currently includes two prospective multicenter cohorts (Development Cohort NCT02457637 and Validation Cohort NCT03641872) of patients with acute exacerbation of chronic liver disease (33, 34). Patients in both cohorts were followed up for more than 1 year. The ethics committee of Renji Hospital and Shiyan Taihe Hospital approved this study.

### Inclusion and Exclusion Criteria

**The inclusion criteria were as follows:**

(1) HBV infection (HBV surface antigen positive ≥ 6 months), a history of abnormal liver function, with or without cirrhosis;
(2) Meeting the EASL-ACLF diagnostic criteria (7):

No ACLF: (1) patients with no organ failure; (2) patients with single “non-kidney” organ failure (i.e., single failure of the liver, coagulation, circulation or respiration) and serum creatinine (Cr) < 1.5 mg/dl and no hepatic encephalopathy (HE); and (3) patients with single cerebral failure and serum Cr < 1.5 mg/dl.

ACLF grade 1: (1) patients with single kidney failure; (2) patients with single failure of the liver, coagulation, circulation or respiration and serum Cr ranging from 1.5 to 1.9 mg/dl and/or HE grade 1 or grade 2; and (3) patients with single cerebral failure and serum Cr ranging from 1.5 to 1.9 mg/dl.

ACLF grade 2: patients with two organ failures of any combination.

ACLF grade 3: patients with three or more organ failures of any combination.

**The exclusion criteria were as follows:**

(1) Hepatocellular carcinoma or other liver malignancies before or during admission.
(2) Extrahepatic malignancies or severe chronic extrahepatic disease.
(3) Age younger than 18 or older than 80 years.
(4) Pregnancy.
(5) HIV infection.

### Data Retrieval

All clinical data for the enrolled patients were extracted from the CATCH-LIFE study database, including demographic data (age, gender) and the patient's cirrhosis status. Laboratory data of the patients at the time of diagnosis of ACLF and on days 4, 7, 14, 21, and 28 included the following: alanine aminotransferase (ALT), aspartate aminotransferase (AST), total bilirubin (Tbil), international normalized ratio (INR), and serum Cr levels; HBV-DNA load; HE grade; ascites; coinfection; and 28-day, 90-day, and 1-year outcomes (death was the outcome event). The frequency, combination pattern, and plasma volume per use of PE-based NB-ALSS therapy were recorded. Clinical data at the time of ACLF diagnosis were used as baseline data. The MELD score of each patient was calculated according to the formula: MELD score = [9.57 × ln(Cr mg/dl) + 3.78 × ln(Tbil mg/dl) + 11.20 × ln(INR) + 6.43 (except for cholestatic and alcoholic liver disease, 6.43 should be added)] (35). The HBV-ACLF patients were divided into four grades according to the correlation between the MELD score and the risk of death (4): MELD grade 1, MELD scores < 20; MELD grade 2, MELD scores of 20 to < 30; MELD grade 3, MELD scores of 30 to < 40; and MELD grade 4, MELD scores ≥ 40. The HBV-ACLF patients were also classified into three types based on cirrhotic status: Type A, HBV-ACLF patients without cirrhosis; Type B, HBV-ACLF patients based on compensatory cirrhosis; and Type C, HBV-ACLF patients based on decompensated cirrhosis (36). In addition, SIRS scores were calculated according to the consensus of the American College of Chest Physicians/Society of Critical Care Medicine Consensus Conference Committee (37).

### Research Grouping

#### The Standard Medical Therapy (SMT) Group

Patients who received SMT alone during hospitalization were enrolled in the SMT group. SMT includes the following: a high-calorie diet; enteral nutrition is recommended; correction of hypoproteinemia; correction of water-electrolyte and acid-base balance; NAs for HBV-DNA-positive patients; anti-infective therapy for infection; a restricted protein diet; lactulose, L-ornithine aspartate for HE; diuretics and tolvaptan for ascites; vasoactive drugs; maintenance of arterial blood pressure and water restriction for hepatorenal syndrome; and oxygen therapy for hepatopulmonary syndrome.

#### The SMT+PE Group

Patients who received at least one PE-based NB-ALSS therapy in addition to SMT during hospitalization were enrolled in the SMT+PE group. PE-based NB-ALSS includes PE alone or in combination with hemofiltration (HF), a double plasma molecular adsorption system (DPMAS), plasma diafiltration (PDF), plasma bilirubin adsorption (PBA), continuous renal replacement therapy (CRRT), or plasma perfusion (PP).

### Statistical Analysis

Normally and non-normally distributed continuous variables are presented as means ± standard deviations (SDs) and medians (interquartile ranges, IQRs), respectively. Categorical variables are shown as *n* (%). To compare differences between two groups, *t*-tests, chi-square tests and Mann-Whitney *U*-tests were used. Propensity score matching analysis was employed to eliminate confounding bias between two groups. The propensity score was calculated according to age, sex, ACLF type, ACLF grade, HE grade, Tbil, serum sodium (Na^+^), Cr, INR, infection, ascites, respiratory failure, and circulatory failure, and the nearest neighbor 1:1 matching scheme was adopted. The 28-day, 90-day, and 1-year survival rates of HBV-ACLF patients were calculated using Kaplan-Meier survival curves. The log-rank test was used to compare survival rates between groups and univariate and multivariate Cox regression analyses were used to determine independent prognostic factors for HBV-ACLF patients and the factors affecting PE-based NB-ALSS efficacy. SPSS software, version 24.0 (SPSS, Inc., Chicago, IL, USA) was used for the data analysis. Figures were produced using GraphPad Prism software, version 6.0 (GraphPad, LLC, San Diego, USA). A two-tailed *p-*value < 0.05 was considered statistically significant.

## Results

### Patient Characteristics

**Figure 1:**
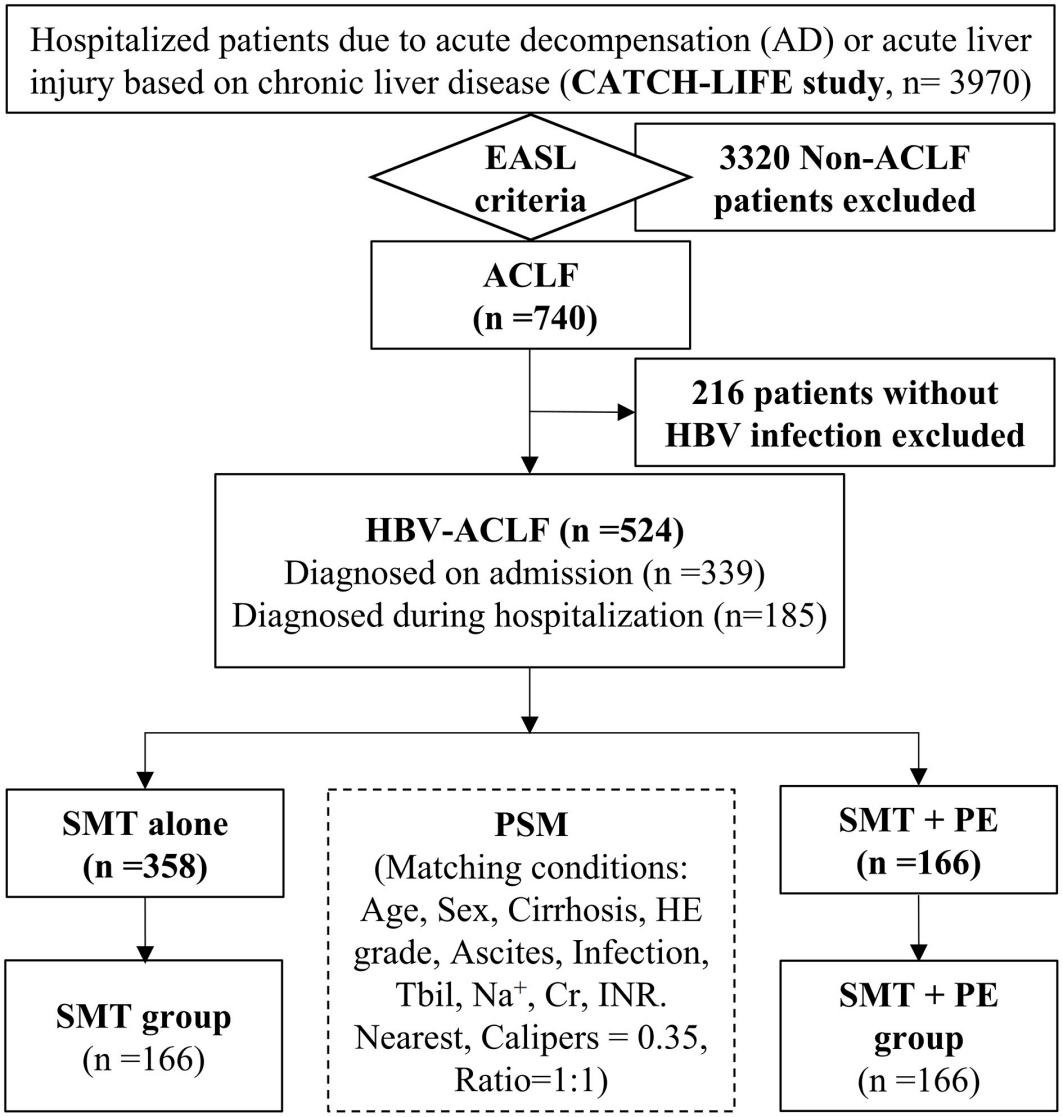
Flow chart of the study. HBV, hepatitis B virus; ACLF, acute-on-chronic liver failure; SMT, standard medical therapy; PE, plasma exchange; PSM, propensity score match; HE, hepatic encephalopathy; Tbil, total bilirubin; Na^+^, serum sodium; Cr, serum creatinine; INR, international normalized ratio; EASL, European Association for The Study of Liver Diseases.

A total of 3,970 patients with chronic liver disease admitted for acute decompensation or acute liver injury were screened for this study. Of them, 740 patients met the EASL-ACLF diagnostic criteria, 216 patients without HBV infection were excluded, and 524 HBV-ACLF patients were ultimately enrolled. Of these, 339 patients were diagnosed with ACLF on admission, and the remaining 185 developed ACLF during hospitalization (Figure 1). Among the 524 HBV-ACLF patients, 440 (83.97%) were male. A total of 115 (21.95%) patients exhibited no evidence of liver cirrhosis (Type A); in contrast, 299 (57.06%) had compensated cirrhosis (Type B), and 110 (20.99%) had decompensated cirrhosis (Type C). Of these, 62 (11.83%), 403 (76.91%), and 59 (11.26%) cases were classified as ACLF grade 1, 2, and 3, respectively. The numbers of patients with MELD grades 1, 2, 3, and 4 were 14 (2.67%), 166 (31.68%), 306 (58.40%), and 38 (7.25%), respectively. A total of 358 patients with HBV-ACLF received SMT alone (SMT group), whereas 166 received PE-based NB-ALSS in addition to SMT (SMT+PE group). The 166 HBV-ACLF patients in the SMT+PE group received a total of 422 PE-based NB-ALSS therapies during hospitalization at least once and up to nine times, with a median of two times. The modes of NB-ALSS therapies included PE alone 294 (69.67%) times, PE + HF 65 (15.40%) times, PE + DPMAS 45 (10.67%) times, and PE + PBA 18 (4.27%) times. A median volume of 2,000 mL fresh frozen plasma (FFP) was used for single PE-based NB-ALSS therapy, and the median time of initiation of PE-based NB-ALSS treatment was 5 days after admission (Table 1).

**Table 1:** Baseline characteristics of HBV-ACLF patients who received SMT alone or SMT+PE.

**Parameters**	**Entire patients**			**Propensity score-matched patients**		
	**SMT+PE** **(***n*** = 166)**	**SMT** **(***n*** = 358)**	***p*** **-value**	**SMT+PE** **(***n*** = 166)**	**SMT** **(***n*** = 166)**	***p-*** **value**
PE times	2.00 (1.00, 3.00)			2.00 (1.00, 3.00)		
Mean plasma dose	2,000 (1,600, 2,800)			2,000 (1,600, 2,800)		
Therapy timing	5.00 (3.00, 8.00)			5.00 (3.00, 8.00)		
**Modes of ALSS**						
PE	294 (69.67%)			294 (69.67%)		
PE + HF	65 (15.40%)			65 (15.40%)		
PE + DPMAS	45 (10.67%)			45 (10.67%)		
PE + PBA	18 (4.27%)			18 (4.27%)		
Age (year)	47.47 ± 11.40	48.31 ± 11.17	0.428	47.47 ± 11.40	47.30 ± 11.35	0.889
Sex (male)	145 (87.35%)	295 (82.40%)	0.151	145 (87.35%)	22 (8.00%)	0.87
HE (≥3)	8 (4.82%)	37 (10.34%)	0.036	8 (4.82%)	17 (10.24%)	0.061
Respiratory failure	10 (6.02%)	28 (7.82%)	0.461	10 (6.02%)	11 (6.63%)	0.813
Circulatory failure	2 (1.20%)	16 (4.47%)	0.056	2 (1.20%)	1 (0.60%)	0.562
Infection (yes)	56 (33.73%)	147 (41.06%)	0.109	56 (33.73%)	56 (33.73%)	1.00
Ascites (yes)	68 (40.96%)	143 (39.94%)	0.825	68 (40.96%)	64 (38.55%)	0.654
ACLF type (A/B/C)	49/98/19	66/201/91	<0.001	49/98/19	45/106/15	0.620
ACLF grade (1/2/3)	10/146/10	52/257/49	<0.001	10/146/10	16/132/18	0.112
MELD grade (1/2/3/4)	1/58/97/10	13/108/209/28	0.157	1/58/97/10	2/45/108/11	0.455
Meld score	31.73 ± 5.37	31.12 ± 5.60	0.241	31.73 ± 5.37	31.66 ± 4.65	0.896
Meld-Na score	32.23 ± 4.97	31.96 ± 5.41	0.589	32.23 ± 4.97	32.23 ± 4.56	0.991
SIRS score	0.81 ± 0.09	0.99 ± 0.07	0.122	0.81 ± 0.09	0.95 ± 0.10	0.318
NH3 (μmol/L)	62.00 (34.60, 83.50)	48.20 (27.90, 80.50)	0.173	62.00 (34.60, 83.50)	48.00 (28.90, 80.50)	0.332
CRP (μg/L)	12.55 (7.78, 17.70)	11.95 (6.49, 22.06)	0.644	12.55 (7.78, 17.70)	12.50 (6.49, 19.90)	0.638
PCT (μg/L)	0.40 (0.00, 0.93)	0.34 (0.00, 0.79)	0.965	0.40 (0.00, 0.93)	0.67 (0.44, 1.17)	0.333
AFP (μg/L)	56.69 (20.09, 185.30)	30.80 (6.89, 108.90)	0.001	56.69 (20.09, 185.30)	36.10 (10.96, 135.30)	0.087
CA199 (μg/L)	54.64 (15.65, 223.70)	56.70 (21.62, 150.59)	0.793	54.64 (15.65, 223.70)	57.44 (26.10, 170.73)	0.93
Log (HBV-DNA)	4.85± 2.09	4.39 ± 2.15	0.034	4.85± 2.09	4.31 ± 2.05	0.030
ALT (U/L)	188.00 (70.65, 607.50)	122.50 (46.98, 385.75)	0.002	188.00 (70.65, 607.50)	186.60 (76.15, 521.00)	0.742
AST (U/L)	192.70 (99.50, 469.00)	151.65 (73.75, 309.58)	0.003	192.70 (99.50, 469.00)	179.00 (104.23, 418.50)	0.519
GGT (U/L)	69.00 (46.25, 101.00)	58.00 (36.00, 88.00)	0.001	69.00 (46.25, 101.00)	61.00 (41.00, 94.00)	0.123
AKP (U/L)	144.65 (111.25, 199.50)	128.50 (98.00, 170.25)	0.002	144.65 (111.25, 199.50)	135.50 (108.00, 170.00)	0.056
Tbil (μmol/L)	437.48 ± 162.20	380.17 ± 188.88	0.001	437.48 ± 162.20	431.35 ± 162.76	0.731
Alb (g/L)	31.79 ± 5.70	30.95 ± 6.18	0.138	31.79 ± 5.70	31.58 ± 5.23	0.723
Na^+^ (mmol/L)	135.76 ± 5.48	134.14 ± 5.87	0.003	135.76 ± 5.48	135.37 ± 4.89	0.498
K^+^ (mmol/L)	3.91 ± 0.64	3.96 ± 0.74	0.481	3.91 ± 0.64	3.95 ± 0.72	0.666
Cr (μmol/L)	83.90 (60.95, 106.18)	80.95 (60.00, 149.33)	0.321	83.90 (60.95, 106.18)	71.10 (56.75, 104.43)	0.079
BUN (mmol/L)	4.44(2.99, 8.02)	6.00 (3.63, 11.96)	0.001	4.44 (2.99, 8.02)	4.88 (3.30, 7.90)	0.475
PT (s)	28.60 (21.00, 33.50)	24.70 (16.30, 31.60)	<0.001	28.60 (21.00, 33.50)	27.20 (16.35, 31.60)	0.008
INR	2.76 (2.53, 3.26)	2.75 (2.39, 3.28)	0.378	2.76 (2.53, 3.26)	2.78 (2.55, 3.41)	0.485
WBC (× 10^9^/L)	8.00 ± 4.17	8.26 ± 4.88	0.562	8.00 ± 4.17	8.24 ± 3.65	0.576
HGB (g/L)	117.22 ± 21.69	109.55 ± 26.10	0.001	117.22 ± 21.69	116.40 ± 24.75	0.750
PLT (× 10^9^/L)	82.50 (54.00, 117.00)	78.50 (50.00, 120.00)	0.507	82.50 (54.00, 117.00)	87.00 (61.70, 131.00)	0.064

*HBV-ACLF, hepatitis B virus-associated acute-chronic liver failure; ACLF type: A, non-cirrhosis; B, compensatory cirrhosis; C, decompensated cirrhosis; NH3, blood ammonia; CRP, c-reactive protein; PCT, procalcitonin; AFP, alpha fetal protein; CA199, cancerantigen199; DNA, deoxyribonucleic acid; SMT, standard medical therapy; PE, plasma exchange; HF, hemofiltration; DPMAS, a double plasma molecular adsorption system; PBA, plasma bilirubin adsorption; HE, hepatic encephalopathy; Meld score, Model for end-stage liver disease score; Meld-Na score, Model for end-stage liver disease with the incorporation of serum sodium score; SIRS, systemic inflammatory response syndrome; ALT, alanine aminotransferase; AST, aspartate aminotransferase; GGT, γ-glutamyltransferase; AKP, alkaline phosphatase; Tbil, total bilirubin; Alb, albumin; Na^+^, serum sodium; K^+^, serum potassium; Cr, serum creatinine; BUN, blood urea nitrogen; PT, prothrombin time; INR, international normalized ratio; WBC, white blood cell; HGB, hemoglobin; PLT, platelet*.

Overall, there were significant differences in baseline characteristics between the patients treated with SMT+PE and SMT alone, such as ACLF type, ACLF grade, alpha fetal protein (AFP), ALT, and AST. For PSM-matched 166 pairs of HBV-ACLF patients, there was no significant difference in baseline data between the SMT+PE group and SMT group, except that longer PT and higher HBV-DNA load were observed in patients in the former compared with the latter (PT, 28.60 vs. 27.20, *p* = 0.008; HBV-DNA, 4.85 log. vs. 4.31 log., *p* = 0.030) (Table 1).

### PE-Based NB-ALSS Significantly Improves the Short-Term Survival Rates of ACLF Patients, Especially in MELD Grade 3 Patients

**Figure 2:**
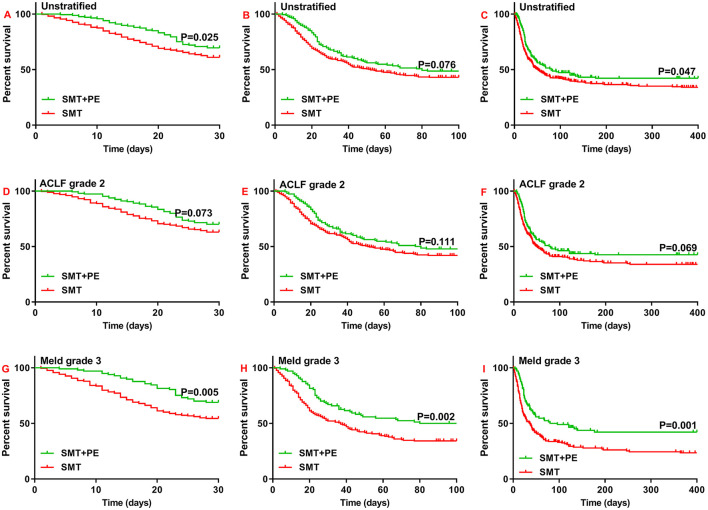
Survival curves for patients with HBV-ACLF who received SMT alone or SMT+PE. **(A–C)** 28-day **(A)**, 90-day **(B)**, and 1-year **(C)** survival curves for patients with HBV-ACLF. **(D–F)** 28-day **(D)**, 90-day **(E)**, and 1-year **(F)** survival curves for HBV-ACLF patients with ACLF grade 2. **(G–I)** 28-day **(G)**, 90-day **(H)**, and 1-year **(I)** survival curves for HBV-ACLF patients with MELD scores of 30–40. SMT, standard medical therapy; PE, plasma exchange; HBV-ACLF, hepatitis B virus-associated acute-on-chronic liver failure.

According to Kaplan-Meier survival analysis, among the enrolled HBV-ACLF patients, the 28-day and 1-year survival rates of patients in the SMT+PE group were significantly higher than those in the SMT group (28-day, 69.50% vs. 60.90%, *p* = 0.025; 1-year, 42.20% vs. 34.10%, *p* = 0.047) (Figures 2A,C). The 90-day survival rate was also higher in patients in the SMT+PE group than in the SMT group, though without a significant difference (48.70% vs. 42.80%, *p* = 0.076) (Figure 2B). Subgroup analysis revealed no significant difference in the survival rate of HBV-ACLF patients between the two groups at ACLF grade 1 and ACLF grade 3 (Supplementary Figures 1G–I). However, for ACLF grade 2 patients, 28-day, 90-day, and 1-year survival rates in the SMT+PE group were higher than those in the SMT group (Figures 2D–F), but the difference was not significant (28-day, 70.10 vs. 62.90%, *p* = 0.073; 90-day, 47.80 vs. 41.60%, *p* = 0.111; 1-year, 42.50 vs. 34.00%, *p* = 0.069). Subgroup analysis according to MELD grade revealed no significant difference in the survival rates of HBV-ACLF patients between the two groups at MELD grade 1, MELD grade 2, and MELD grade 4 (Supplementary Figures 1A–F); for MELD grade 3 patients, 28-day, 90-day, and 1-year survival rates were significantly higher in patients in the SMT+PE group than in the SMT group (28-day, 68.90 vs. 54.10%, *p* = 0.005; 90-day, 50.00 vs. 33.80%, *p* = 0.002; 1-year, 42.20 vs. 23.50%, *p* = 0.001) (Figures 2G–I).

**Figure 3:**
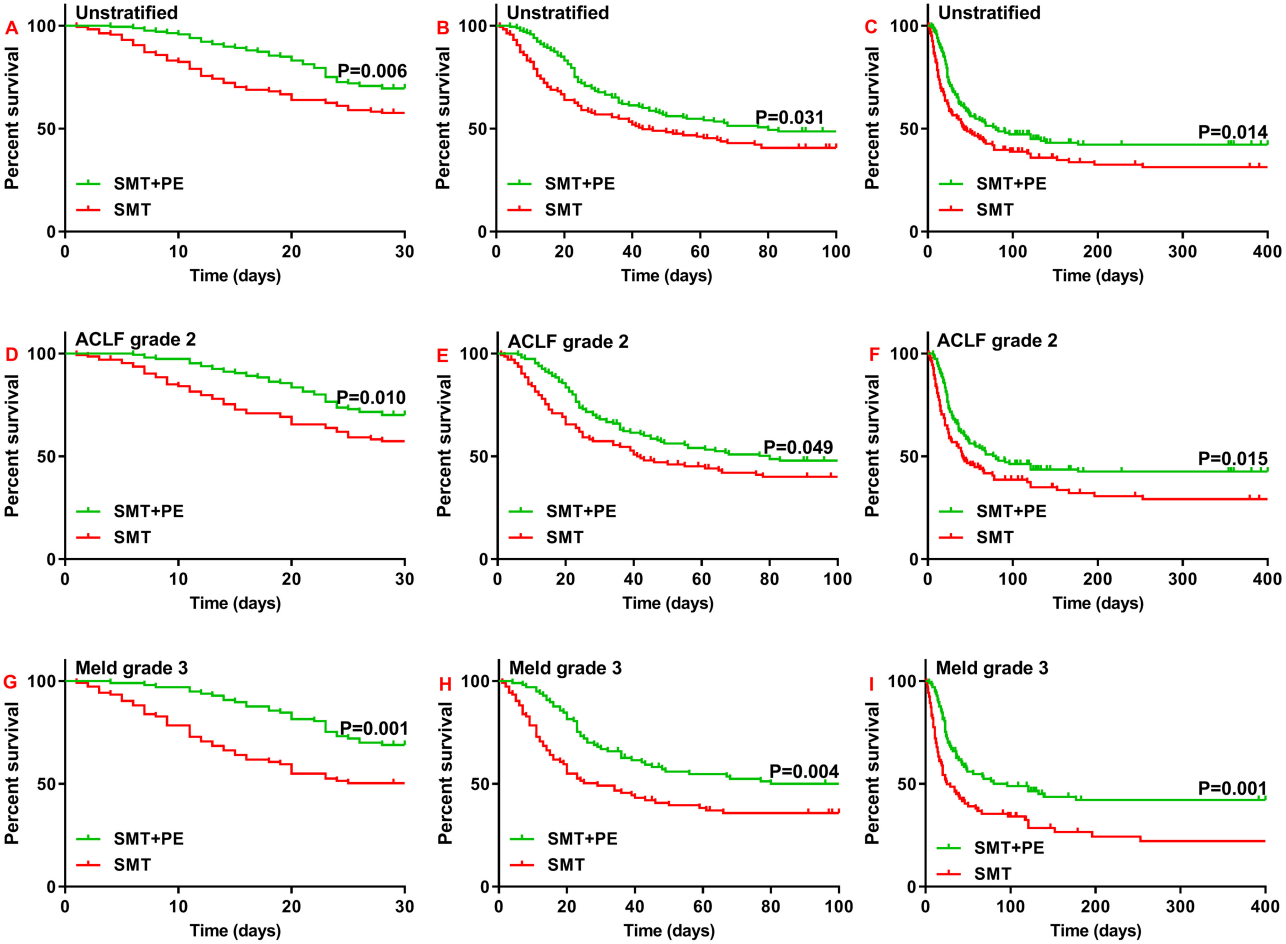
Survival curves for 166 pairs of matched patients with HBV-ACLF who received SMT alone or SMT+PE. **(A–C)** 28-day **(A)**, 90-day **(B)**, and 1-year **(C)** survival curves for patients with HBV-ACLF. **(D–F)** 28-day **(D)**, 90-day **(E)**, and 1-year **(F)** survival curves for HBV-ACLF patients with ACLF grade 2. **(G–I)** 28-day **(G)**, 90-day **(H)**, and 1-year **(I)** survival curves for HBV-ACLF patients with MELD scores of 30 to 40. PE, plasma exchange; SMT, standard medical therapy; HBV-ACLF, hepatitis B virus-associated acute-on-chronic liver failure.

Importantly, in 166 pairs of PSM-matched HBV-ACLF patients, 28-day, 90-day, and 1-year survival rates were all significantly higher in the SMT+PE group than in the SMT group (28-day, 69.50 vs. 57.60%, *p* = 0.006; 90-day, 48.70 vs. 40.70%, *p* = 0.031; 1-year, 42.20 vs. 31.30%, *p* = 0.014) (Figures 3A–C). Moreover, subgroup analysis based on ACLF and MELD grades showed significantly higher 28-day, 90-day, and 1-year survival rates for the SMT+PE group among patients with ACLF grade 2 (Figures 3D–F) and MELD grade 3 (Figures 3G–I), especially for the latter. In addition, 28-day, 90-day, and 1-year survival rates in patients in the SMT+PE group were 18.60, 14.20, and 20.10% higher than those in the SMT group, respectively (Table 2). However, no significant difference was found in the short-term survival rate between the SMT group and SMT+PE group of HBV-ACLF patients with ACLF grade 1, ACLF grade 3, MELD grade 2, and MELD grade 4 (Supplementary Figure 2).

**Table 2:** Cumulative survival rates of HBV-ACLF patients who received SMT alone or combination therapy with SMT and PE.

		**Time**	**Survival rate**		**χ^**2**^ value**	***p*** **-value**	**Survival rate difference**
			**SMT+PE**	**SMT**			
Unstratified	All patients	28 days	69.50%	60.90%	5.005	0.025	8.60%
		90 days	48.70%	42.80%	3.149	0.076	5.90%
		1 year	42.20%	34.10%	3.944	0.047	8.10%
	Matched patients	28 days	69.50%	57.60%	7.508	0.006	11.90%
		90 days	48.70%	40.70%	4.633	0.031	8.00%
		1 year	42.20%	31.30%	5.991	0.014	10.90%
ACLF grade 2	All patients	28 days	70.10%	62.90%	3.219	0.073	7.20%
		90 days	47.80%	41.60%	2.537	0.111	6.20%
		1 year	42.50%	34.00%	3.312	0.069	8.50%
	Matched patients	28 days	70.10%	57.30%	6.637	0.010	12.80%
		90 days	47.80%	40.00%	3.873	0.049	7.80%
		1 year	42.50%	29.20%	5.940	0.015	13.30%
Meld grade 3	All patients	28 days	68.90%	54.10%	7.914	0.005	14.80%
		90 days	50.00%	33.80%	9.253	0.002	16.20%
		1 year	42.20%	23.50%	11.349	0.001	18.70%
	Matched patients	28 days	68.90%	50.30%	10.562	0.001	18.60%
		90 days	50.00%	35.80%	6.637	0.004	14.20%
		1 year	42.20%	22.10%	11.229	0.001	20.10%

*HBV-ACLF, hepatitis B virus-associated acute-on-chronic liver failure; ACLF, acute-on-chronic liver failure; PE, plasma exchange; SMT, standard medical therapy; Meld score, Model for end-stage liver disease score*.

### PE-Based NB-ALSS Does Not Lead to Continuous Improvement in the Laboratory Parameters of HBV-ACLF Patients Within 28 Days

**Figure 4:**
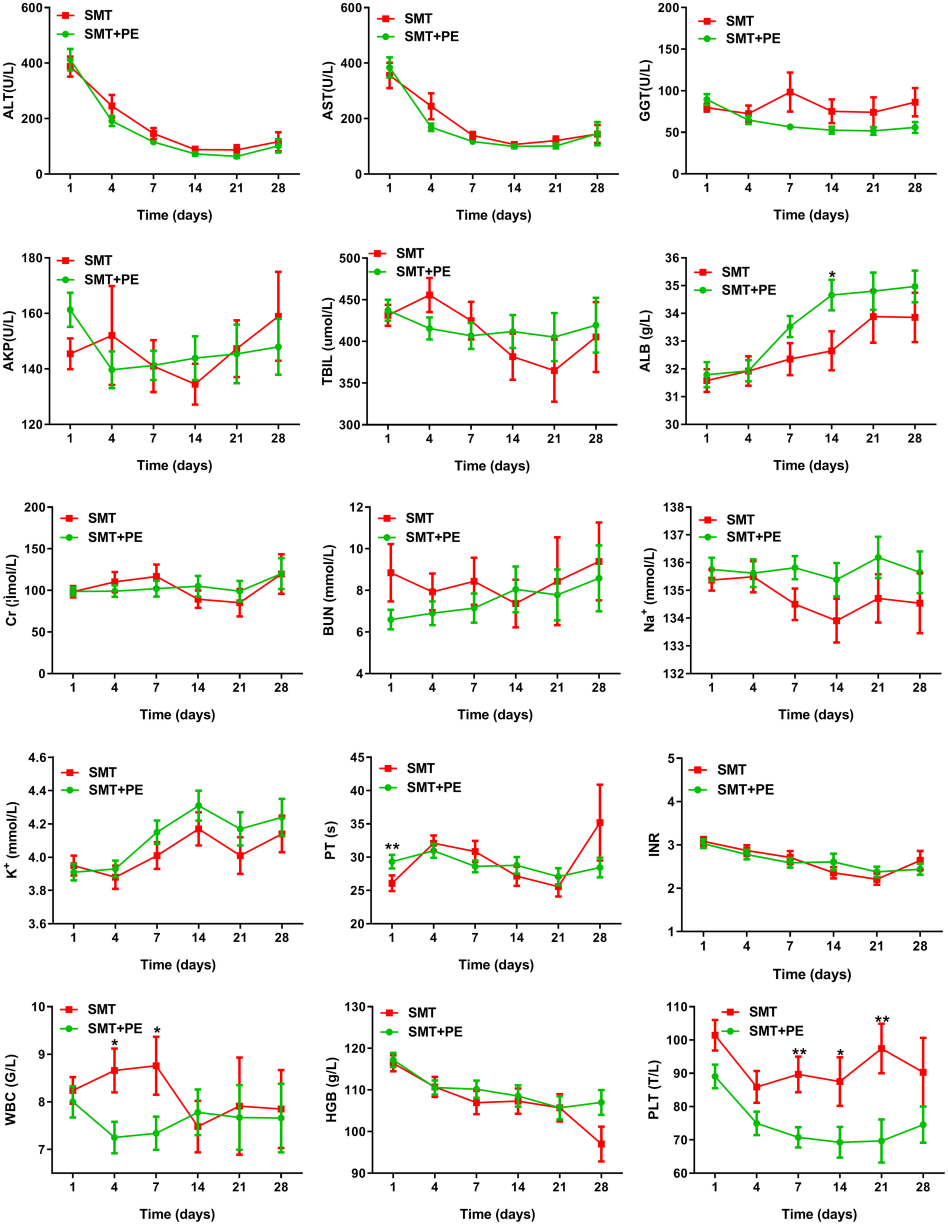
Changes in laboratory parameters in HBV-ACLF patients in the SMT group and SMT+PE group. SMT, standard medical therapy; PE, plasma exchange; ALT, alanine aminotransferase; AST, aspartate aminotransferase; GGT, γ-glutamyltransferase; AKP, alkaline phosphatase; Tbil, total bilirubin; Alb, albumin; Na^+^, serum sodium; K^+^, serum potassium; Cr, serum creatinine; BUN, blood urea nitrogen; PT, prothrombin time; INR, international normalized ratio; WBC, white blood cell; HGB, hemoglobin; PLT, platelet; ^*^P value < 0.05; ^**^P value < 0.01.

Among 166 pairs of PSM-matched HBV-ACLF patients, significant reductions in Tbil, GGT, and PT were observed only on day 7 in those treated with SMT+PE compared with those treated with SMT (Table 3). Although ALT, AST, and GGT levels in the SMT+PE group were lower in patients treated with SMT+PE on days 4, 7, 14, 21, and 28, and thereafter, the difference was not significant (Figure 4). The alkaline phosphatase (AKP) level of HBV-ACLF patients in the SMT+PE group showed a continuous decline but fluctuated in patients in the SMT group. However, Alb displayed a gradually increasing trend in both groups, and Alb was significantly higher in the SMT+PE group than in the SMT group on day 14. There were no significant differences in Cr, blood urea nitrogen (BUN), Na^+^, serum potassium (K^+^), or hemoglobin (HGB) levels at each time point. Although the WBC count was significantly lower in the SMT+PE group than in the SMT group on day 4 and 7, the PLT count was significantly lower in the former on days 7, 14 and 21 (Figure 4). Further analysis in SIRS scores showed that SIRS scores were significantly lower in the SMT+PE group than those in the SMT group on day 7 (0.62 vs. 1.03, *p* = 0.002) and day 14 (0.64 vs. 1.01, *p* = 0.035) (Supplementary Figure 3).

**Table 3:** Changes in laboratory parameters in patients with HBV-ACLF who received SMT alone or combination therapy with SMT and PE-based NB-ALSS.

**Parameter**	**Group**	**Mean**	**SE**	***t*** **-value**	***p*** **-value**
ΔTbil	SMT	21.38	17.12	2.039	0.043
	SMT+PE	−22.19	13.20		
ΔGGT	SMT	20.22	22.84	2.266	0.025
	SMT+PE	−26.12	7.07		
ΔPT	SMT	8.09	1.98	3.795	<0.001
	SMT+PE	0.48	0.97		

Δ, *the difference in the value between day 7 and the baseline*.

*GGT, γ-glutamyltransferase; Tbil, total bilirubin; PT, prothrombin time*.

### PE-Based NB-ALSS Is the Only Independent Protective Factor for Short-Term Prognosis in Patients With HBV-ACLF

Based on multivariate Cox regression analysis, PE-based NB-ALSS was the only independent protective factor for the survival of HBV-ACLF patients at 28 days, 90 days, and 1 year (28 days, HR = 0.516, *p* = 0.001; 90 days, HR = 0.663, *p* = 0.010; 1 year, HR = 0.610, *p* = 0.051). Advanced age, combined with cerebral failure (HE ≥ 3), respiratory failure and high MELD score were independent risk factors for survival at 28 days (Table 4). The independent risk factors for 90-day and 1-year survival of HBV-ACLF patients are shown in Table 4. The results of univariate Cox regression analysis are shown in Supplementary Table 1.

**Table 4:** Prognostic factors at 28 days, 90 days, and 1 year in HBV-ACLF patients treated with SMT alone or in combination with SMT and PE-based NB-ALSS.

**Multivariate**	***p*** **value**	**HR**	**95% CI HR**		**Time point**
**Parameter**			**Lower bound**	**Upper bound**	
PE	0.001	0.516	0.353	0.755	28 days
Age	<0.001	1.05	1.032	1.068	
HE(≥3)	<0.001	3.774	2.208	6.452	
Respiratory failure	<0.001	3.645	1.955	6.798	
MELD score	<0.001	1.09	1.058	1.122	
PE	0.006	0.647	0.473	0.884	90 days
Age	<0.001	1.049	1.035	1.064	
HE(≥3)	<0.001	3.945	2.349	6.625	
Respiratory failure	0.001	2.633	1.508	4.596	
Na^+^	0.002	0.949	0.919	0.98	
INR	<0.001	1.325	1.214	1.446	
PE	0.051	0.610	0.372	1.002	1 year
Age	<0.001	1.038	1.016	1.059	
HE(≥3)	0.006	3.221	1.392	7.450	
INR	<0.001	1.289	1.134	1.466	
CRP	0.004	1.017	1.005	1.028	

*HBV-ACLF, hepatitis B virus-associated acute-on-chronic liver failure; PE, plasma exchange; HE, hepatic encephalopathy; Meld score, Model for end-stage liver disease score; Na^+^, serum sodium; INR, international normalized ratio; CRP, c-reactive protein; HR, hazard ratio*.

### Frequency Is an Important Factor Affecting the Efficacy of PE-Based NB-ALSS

Multivariate Cox regression analysis showed that for HBV-ACLF patients treated with PE-based NB-ALSS, the frequency of the therapy was the only independent protective factor for 28-day and 90-day survival (28-day, HR = 0.597, *p* = 0.001; 90-day, HR = 0.772, *p* = 0.018). Respiratory failure and high INR were independent risk factors for 28-day survival, and advanced age, respiratory failure, high BUN and high INR were independent risk factors for 90-day prognosis (Table 5). The results of univariate Cox regression analysis are shown in Supplementary Table 2.

**Table 5:** Prognostic factors in HBV-ACLF patients treated with PE-based NB-ALSS.

**Multivariate**	***p*** **-value**	**HR**	**95% CI HR**		**Time point**
**Parameters**			**Lower bound**	**Upper bound**	
PE times	0.001	0.597	0.436	0.816	28 days
Respiratory failure	0.001	4.497	1.843	10.973	
INR	0.007	1.216	1.056	1.400	
PE times	0.018	0.772	0.623	0.957	90 days
Age	0.003	1.033	1.011	1.055	
Respiratory failure	0.022	2.829	1.159	6.905	
BUN	0.035	1.037	1.003	1.072	
INR	0.002	1.225	1.075	1.396	
Respiratory failure	0.057	3.510	0.965	12.773	1 year
CRP	0.001	1.024	1.010	1.039	
PT	<0.001	1.039	1.018	1.061	

*PE, plasma exchange; HE, hepatic encephalopathy; INR, international normalized ratio; BUN, blood urea nitrogen; CRP, c-reactive protein; PT, prothrombin time; HR, hazard ratio*.

## Discussion

In this study, PE-based NB-ALSS significantly improved 28-day and 90-day outcomes, as well as 1-year outcomes, in HBV-ACLF patients. Nevertheless, subgroup analysis showed that not all patients with HBV-ACLF benefited from PE-based NB-ALSS. Indeed, PE-based NB-ALSS had significant efficacy only in those with ACLF grade 2 or MELD grade 3, but did not lead to continuous improvement in the laboratory parameters, such as Tbil, PT, GGT, AST, and ALT, of HBV-ACLF patients within 28 days. However, multivariate Cox regression analysis indicated that PE-based NB-ALSS was the only independent protective factor for 28-day and 90-day prognosis in HBV-ACLF patients and that advanced age, cerebral failure, respiratory failure, and high MELD score were independent risk factors for 28-day prognosis. Additionally, advanced age, cerebral failure, respiratory failure, hyponatremia, and elevated INR were independent risk factors for 90-day prognosis, which is consistent with the results of previous studies (22, 25, 26, 38). PE-based NB-ALSS therapy was also found to be an independent protective factor for the 1-year prognosis of HBV-ACLF patients in this study.

Studies in Europe have shown that the molecular adsorbent recirculating system (MARS) can improve the short-term survival rate of patients with ACLF grades 2-3 (39). Ning Qin et al. reported that plasma perfusion (PP) combined with PE therapy significantly improves the short-term outcomes of HBV-ACLF patients, with the best efficacy for those with ACLF grades 2-3 (38). In this study, however, PE-based NB-ALSS therapy only exhibited significant efficacy for ACLF grade 2 patients, which may not only be due to the different therapeutic mechanisms of MARS and PE but also to the different disease characteristics of ACLF patients in the East and West. In fact, alcoholism and HCV infection are the most common causes of ACLF in Western countries, and renal function damage is very common in these ACLF patients, whereas HBV infection is the most common cause of ACLF in China (1, 3, 5, 40). Overall, HBV-ACLF patients are more prone to liver failure and coagulation failure but less prone to renal failure and cerebral failure (5, 6, 38). Of the 332 HBV-ACLF patients enrolled in the matched analysis in this study, 278 (83.73%) had ACLF grade 2; among them, 243 (87.41%) patients had liver failure combined with coagulation failure, 26 (7.83%) had ACLF grade 1, and 28 (8.43%) had ACLF grade 3. Patients with ACLF grade 1 mostly experience renal failure or cerebral failure with renal dysfunction. FFP is rich in coagulation factors, albumin, immunoglobulin and other essential substances and can partially replace the synthesis and detoxification functions of the liver, thereby improving liver and coagulation function in HBV-ACLF patients. Hence, PE-based NB-ALSS has a significant therapeutic effect on patients with EASL-ACLF grade 2. Although PE therapy is effective in clearing albumin-bound toxins, it cannot effectively remove water-soluble toxins of small and medium sizes. Therefore, PE therapy is not ideal for improving renal function in HBV-ACLF patients. In this study, PE-based NB-ALSS therapy showed no significant effect in ACLF grade 1 patients, which may be related to the small number of ACLF grade 1 patients and the weak effect of PE on renal failure. For patients with ACLF grade 1, CRRT or DPMAS in addition to SMT is recommended, as is the combination of PE with CRRT or DPMAS. Patients with ACLF grade 3 underwent at least 3 organ failures, and their 28-day mortality can be as high as 78.6% without LT (7). Although studies have shown that PE can control the occurrence and development of multiple organ failure in ACLF patients, PE therapy may be ineffective for those who have already experienced multiple organ failure. Instead, it will increase the risk of infection, hemodynamic instability and HE (22, 38). In the present study, PE-based NB-ALSS therapy did not improve the outcomes of ACLF grade 3 patients, and it is recommended that such patients receive LT as soon as possible.

Among HBV-ACLF patients with MELD scores between 30 and 40, 28-day, 90-day, and 1-year survival rates increased by 14–20% in patients who received SMT+PE therapy compared with those who received SMT alone. However, in HBV-ACLF patients with MELD scores >40, SMT+PE therapy failed to improve short-term survival rates, similar to the result of Yu et al. (26). Because the MELD scores of the ACLF patients enrolled in Yu et al.'s study were all >30, their study did not investigate the effect of PE therapy in those with MELD scores <30. Among the HBV-ACLF patients included in the matching analysis in the present study, there were only 3 with MELD scores <20, which is too small for a statistical analysis. Our study cohort comprised 45 patients in the SMT group and 58 in the SMT+PE group with MELD scores between 20 and 30, and PE-based NB-ALSS therapy did not improve the short-term survival rate of these patients. However, Xia et al.'s study showed that PE-based NB-ALSS can significantly improve the short-term outcomes of HBV-ACLF patients with a MELD score ≤ 30. In particular, the 12-week survival rate of patients with MELD score ≤ 20 increased by 21%; for patients with MELD score >30, the 4–48 weeks survival rate was significantly lower in those receiving PE therapy than in those receiving SMT (41). Although the sample size (787 cases) was sufficient, Xia et al.'s study was a single-center retrospective study without PSM analysis, and the study by Yu et al. was a prospective randomized controlled study with a higher level of evidence. Therefore, PE-based NB-ALSS therapy can improve short-term outcomes in patients with HBV-ACLF but is likely to have significant efficacy in only a subset of patients. A large, prospective, multicenter randomized controlled study is needed to further define the subgroup of patients who will potentially benefit from PE-based NB-ALSS therapy.

In the study of Chen et al., PE therapy was found to significantly reduce Tbil and INR in ACLF patients (42). Yao et al. also reported that the ALT, AST, Tbil, and INR of HBV-ACLF patients were significantly reduced after PE therapy (43). In our study, there was an instantaneous and significant decrease in Tbil after PE-based NB-ALSS therapy (data not shown), but Tbil and other laboratory parameters rebounded to high levels in the absence of subsequent PE-based NB-ALSS therapy. Indeed, significantly reductions in Tbil, GGT, and PT were only detected on day 7 in PSM-matched HBV-ACLF patients who received PE-based NB-ALSS therapy. According to the consensus of the Asian Pacific Association for the Study of the Liver (APASL), patients with ACLF who develop SIRS within 7 days after disease onset will subsequently develop multiple organ failure (MOF) and are deemed to have a very poor prognosis. Therefore, the development of SIRS within 1 week is an important determinant of prognosis in patients with ACLF (44). PE therapy can significantly reduce levels of pro-inflammatory cytokines in ACLF patients, thereby preventing overactivation of the immune system (22–24, 38, 45). In this study, the WBC count showed a slight decline in the SMT+PE group and an increase in the SMT group. The WBC count in the SMT+PE group was significantly lower than that in the SMT group on day 4 and 7, and the SIRS scores were also significantly lower in the SMT+PE group than in the SMT group on day 7 and 14. Thus, PE-based NB-ALSS therapy reduces the inflammatory response degree in HBV-ACLF patients, which may reduce the occurrence of SIRS and MOF. This finding may explain why in this study, PE-based NB-ALSS therapy improved the prognosis of HBV-ACLF patients without continuous improvement in laboratory parameters. In addition, the disease course of ACLF patients who survive for 28 days usually exceeds 60 days, and most patients begin to recover after 28 days. Nonetheless, the laboratory parameters of HBV-ACLF patients were only recorded for 28 days in this study. Overall, extending the observation period may reveal the effect of PE-based NB-ALSS therapy in improving the laboratory parameters of HBV-ACLF patients.

The platelet count of HBV-ACLF patients receiving PE-based NB-ALSS therapy continued to decrease and was significantly lower than that of patients in the SMT group. This phenomenon may be related to thrombocytopenia caused by the use of heparin (46, 47). Therefore, platelet changes and the coagulation function of ACLF patients receiving PE-based NB-ALSS therapy should be closely observed in the clinic. It is suggested that HBV-ACLF patients with platelet counts <50 × 10^9^/L should be given low molecular weight heparin or citrate as an anticoagulant during the PE-based NB-ALSS process to avoid heparin-induced thrombocytopenia.

In this study, the median PE-based NB-ALSS therapy frequency was 2, and multivariate Cox regression analysis showed that the frequency of the therapy was the only independent protective factor for 28-day and 90-day prognosis in HBV-ACLF patients treated with this modality. This result suggests that the more PE-based NB-ALSS therapy is administered, the better is the prognosis of HBV-ACLF patients. Surprisingly, the timing of PE-based NB-ALSS therapy in HBV-ACLF patients and the volume of FFP used per session were not significantly associated with prognosis. As most of the patients in this study received PE-based NB-ALSS therapy within 1 week after admission and the FFP volume used for each session was ~2 L for the vast majority of patients, we were unable to evaluate the impact of the timing and FFP volume on the efficacy of the therapy. In a multicenter prospective study by Larsen FS et al., high-volume (8–12 L) PE therapy once daily for 3 consecutive days significantly improved outcomes in patients with ALF (22), and Stahl et al.'s showed that low-volume (2–3 L) PE per day was as effective as high-volume PE for ALF patients (48). These results suggest that frequent PE therapy can improve the outcomes of those with liver failure when the FFP volume is no <2 L. In this study, the frequency of PE-based NB-ALSS therapy, rather than the FFP volume, exhibited a significant impact on the therapeutic effect of the therapy, which again confirmed the results of Stahl et al.'s study. Therefore, frequent low-volume PE-based NB-ALSS therapy is recommended for HBV-ACLF patients, potentially reducing both the amount of FFP and the risk of complications such as internal environment disorder and hemodynamic instability.

The CATCH-LIFE study consisted of two prospective multicenter cohorts of acute exacerbations of chronic liver disease established by 15 tertiary hospitals. The locations of these 15 tertiary hospitals cover 95% of the population distribution in China. The HBV-ACLF patients enrolled in this study were all from CATCH-LIFE, effectively reducing selection bias and rendering the research results reliable. Nevertheless, this study has some limitations. First, this was an observational study; the timing, frequency and FFP volume of PE therapy were not carried out uniformly, and the sample size was not large enough. The optimal timing, frequency and FFP volume of PE therapy still need to be determined through a multicenter randomized controlled study. Second, only HBV-ACLF patients were enrolled, and whether the results are applicable to ACLF patients with other etiologies needs further investigation. Third, as this study found that PE-based NB-ALSS therapy could not continuously improve the laboratory parameters of HBV-ACLF patients during the 28-day observation period, the clinical observation period should be extended to further clarify the improvement effect. Finally, this study did not detect levels of inflammatory cytokines before and after PE-based NB-ALSS treatment, and thus the mechanism of PE-based NB-ALSS was not examined. Fortunately, proteomics and metabolomics studies are being conducted using blood samples from this cohort, and the results may clarify the mechanism of PE-based NB-ALSS as ACLF therapy.

In conclusion, this study showed that PE-based NB-ALSS therapy significantly improves the short-term (28/90-day and 1-year) outcomes of patients with HBV-ACLF, and that patients with EASL-ACLF grade 2 or MELD scores of 30–40 are more likely to benefit, thereby reducing their dependence on LT. Moreover, increasing the frequency of PE therapy may provide greater benefits to patients with HBV-ACLF.

## Data Availability Statement

The raw data supporting the conclusions of this article will be made available by the Chinese Chronic Liver Failure (CLIF) Consortium, available at: aclf_group@163.com.

## Ethics Statement

The studies involving human participants were reviewed and approved by the Ethics Committee of Renji Hospital and Shiyan Taihe Hospital. The patients/participants provided their written informed consent to participate in this study.

## Author Contributions

Y-yC and Z-jM were responsible for the study concept, design, and drafted the manuscript. HL (2nd author), X-bW, YH, Z-jM, Y-hG, Z-pQ, FL, X-bL, JS, HL (13th author), S-yW, and Y-hZ performed the data acquisition. All authors had access to the data and a role in writing the manuscript, offered critical revision, and approved the final draft of the manuscript.

## Chinese (Acute ON) Chronic Liver Failure Consortium (Ch-CLIF. C)

Department of Gastroenterology, Ren Ji Hospital, School of Medicine, Shanghai Jiao Tong University—Shan Yin, Wenyi Gu, Yan Zhang, Tongyu Wang, Dandan Wu, Fuchen Dong, Bo Zeng, Liuying Chen, Shijin Wang; Centre of Integrative Medicine, Beijing Ditan Hospital, Capital Medical University—Qun Zhang, Yixin Hou, Yuxin Li, Yunyi Huang; Department of Infectious Diseases, Southwest Hospital, Third Military Medical University (Army Medical University)—Shuning Sun, Wenting Tan, Xiaomei Xiang, Yunjie Dan, Guohong Deng; Department of Infectious Disease, Hunan Key Laboratory of Viral Hepatitis, Xiangya Hospital, Central South University—Jun Chen, Chengjin Liao, Xiaoxiao Liu; Department of Infectious Diseases, Institute of Infection and Immunology, Union Hospital, Tongji Medical College, Huazhong University of Science and Technology—Jing Liu, Ling Xu, Shue Xiong, Yan Xiong, Congcong Zou; Hepatology Unit, Department of Infectious Diseases, Nanfang Hospital, Southern Medical University—Jinjun Chen, Congyan Zhu; Department of Hepatology, First Hospital of Jilin University—Chang Jiang, Xiaoyu Wen, Na Gao, Chunyan Liu; Department of Infectious Disease, Taihe Hospital, Hubei University of Medicine—Qing Lei, Sen Luo; Department of Infectious Disease, The First Hospital of Zhejiang University—Haotang Ren; Department of Liver Intensive Care Unit, Shanghai Public Health Clinical Centre, Fudan University—Xue Mei, Jiefei Wang, Liujuan Ji; Department of Infectious Diseases and Hepatology, Second Hospital of Shandong University—Tao Li, Xuanqiong Fang, Jing Li, Ziyu Wang; Liver Disease Centre, First Affiliated Hospital of Xinjiang Medical University—Rongjiong Zheng, Fangrong Jie, Nan Li; Department of Infectious Disease, Henan Provincial People's Hospital—Huiming Jin; Infectious Disease Center, Affiliated Hospital of Logistics University of People's Armed Police Force—Hai Li, Qing Zhang, Xuequn Zheng; Department of Infectious Disease, Fuzhou General Hospital of Nanjing Military Command—Shaoyang Wang, Taofa Lin.

## Funding

This work was supported by the National Science and Technology Major Project (Grant Nos. 2018ZX10723203 and 2018ZX10302206), the Foundation for Innovative Research Groups of Hubei Provincial Natural Science Foundation (2018CFA031), Hubei Province's Outstanding Medical Academic Leader Program, Project of Hubei University of Medicine (FDFR201902, 2020XGFYZR05, and YC2019006), and Scientific and Technological Project of Shiyan City (20Y08).

## Conflict of Interest

The authors declare that the research was conducted in the absence of any commercial or financial relationships that could be construed as a potential conflict of interest.

## Publisher's Note

All claims expressed in this article are solely those of the authors and do not necessarily represent those of their affiliated organizations, or those of the publisher, the editors and the reviewers. Any product that may be evaluated in this article, or claim that may be made by its manufacturer, is not guaranteed or endorsed by the publisher.
