# Supplementary figures and images for: Plasma Exchange-Based Non-bioartificial Liver Support System Improves the Short-Term Outcomes of Patients With Hepatitis B Virus-Associated Acute-on-Chronic Liver Failure: A Multicenter Prospective Cohort Study
Source: Front Med (Lausanne). 2021 Nov 16;8:779744. doi: 10.3389/fmed.2021.779744 (PMC8635207; doi:10.3389/fmed.2021.779744)

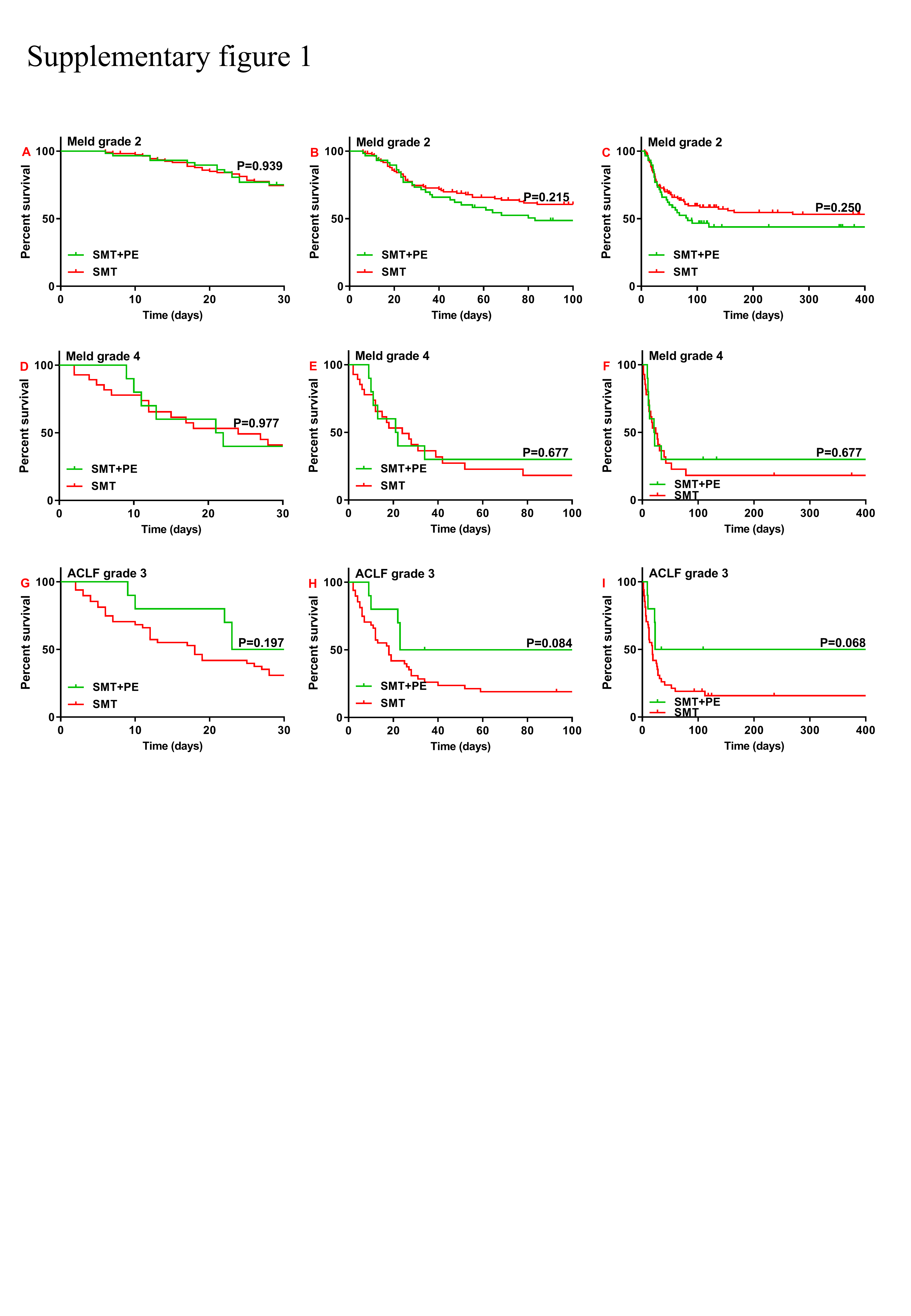

Supplement: Supplementary file 1 [file Image_1.TIF]

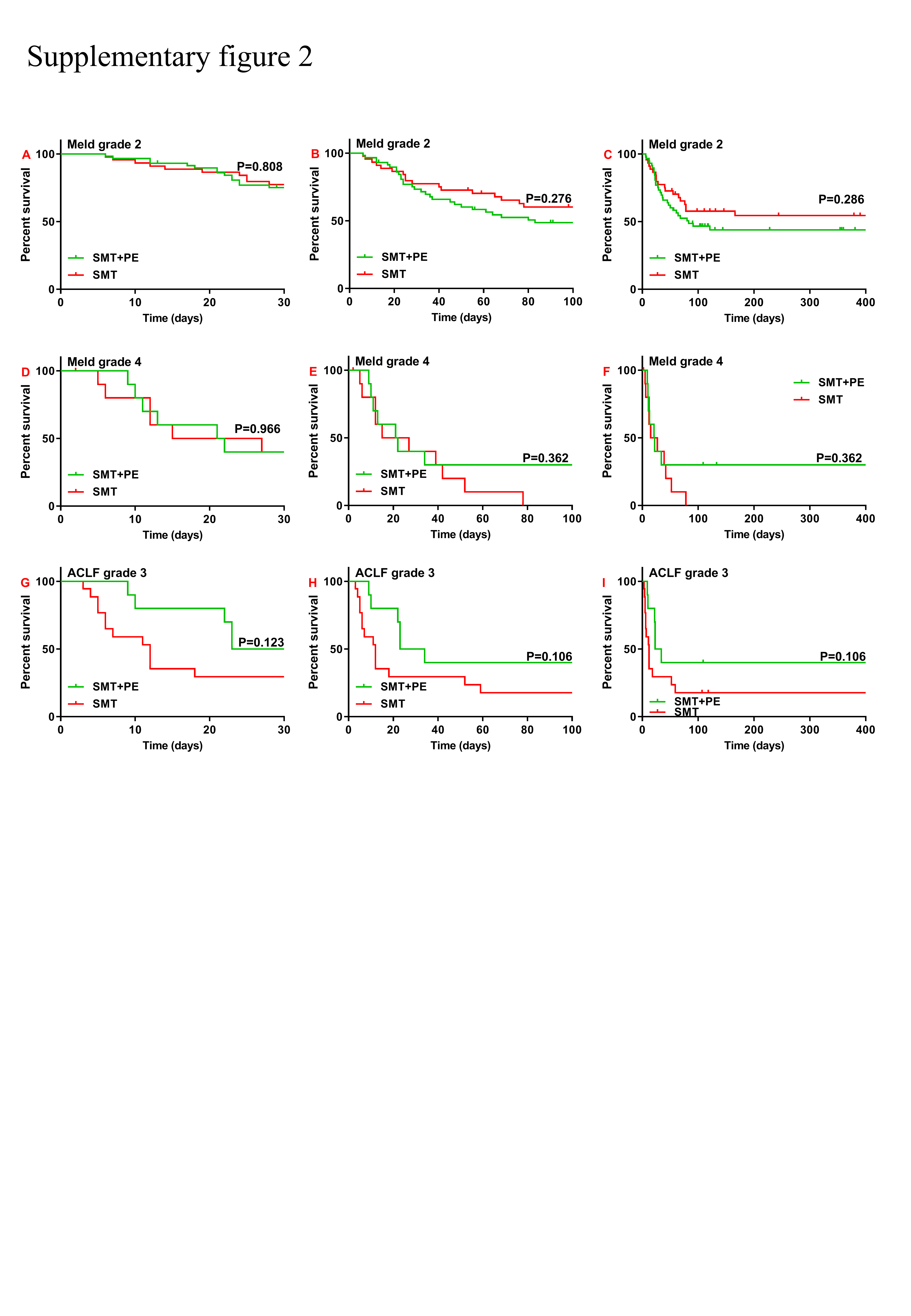

Supplement: Supplementary file 2 [file Image_2.TIF]

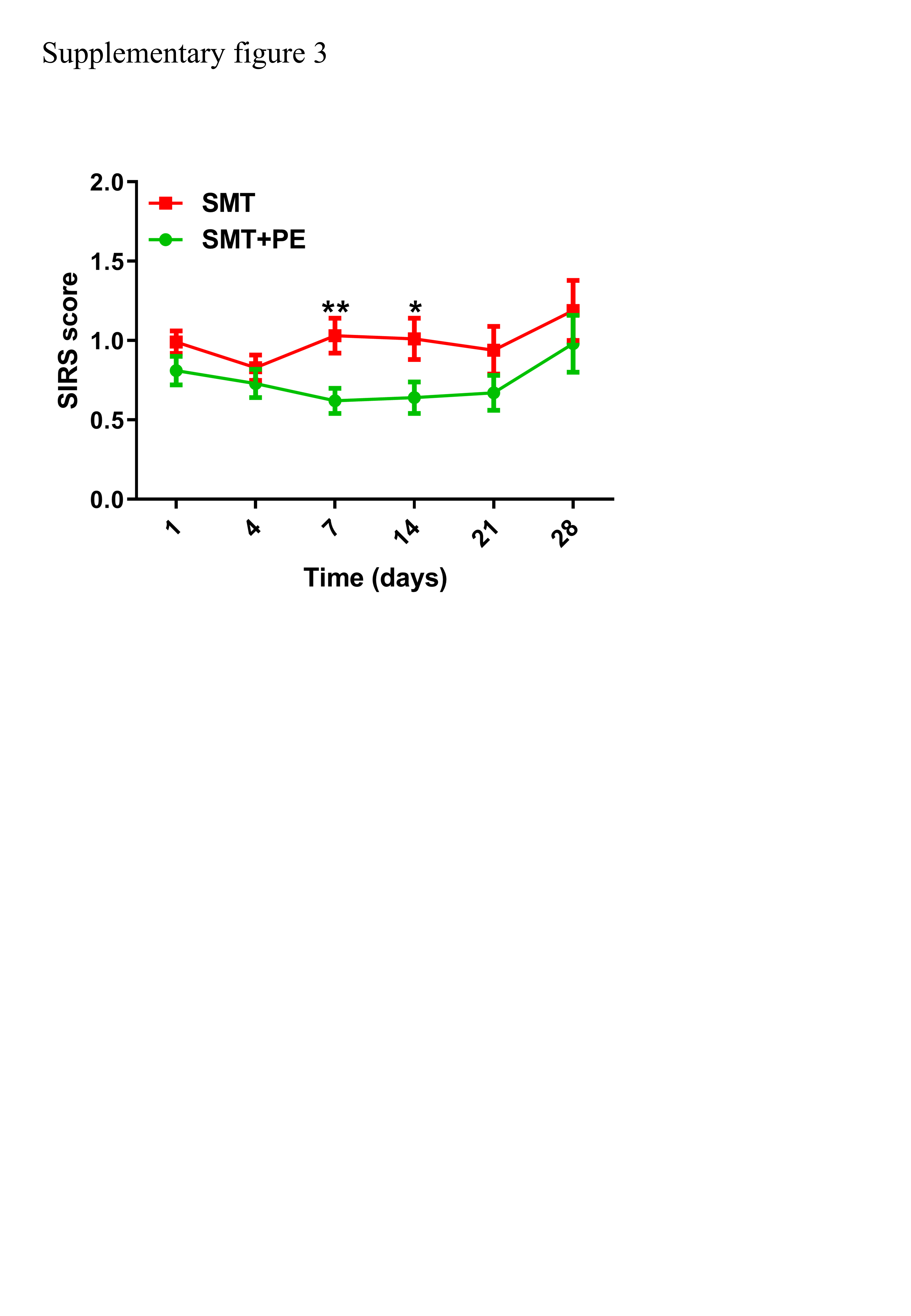

Supplement: Supplementary file 3 [file Image_3.TIF]
