# Supplementary material for: Plasma Exchange-Based Non-bioartificial Liver Support System Improves the Short-Term Outcomes of Patients With Hepatitis B Virus-Associated Acute-on-Chronic Liver Failure: A Multicenter Prospective Cohort Study
Source: Front Med (Lausanne). 2021 Nov 16;8:779744. doi: 10.3389/fmed.2021.779744 (PMC8635207; doi:10.3389/fmed.2021.779744)
Supplement: Supplementary file 4 [file Data_Sheet_1.docx]

**Supplementary figure legends.**

**Fig. S1. Survival curves for patients with HBV-ACLF who received SMT alone or SMT+PE.** **A-C**. 28-day (A), 90-day (B), and 1-year (C) survival curves for HBV-ACLF patients with MELD grade 2. **D-F**. 28-day (D), 90-day (E), and 1-year (F) survival curves for HBV-ACLF patients with MELD grade 4. **G-I**. 28-day (G), 90-day (H), and 1-year (I) survival curves for HBV-ACLF patients with ACLF grade 3. SMT, standard medical therapy; PE, plasma exchange; HBV-ACLF, hepatitis B virus-associated acute-on-chronic liver failure.

**Fig. S2. Survival curves for 166 pairs of matched patients with HBV-ACLF who received SMT alone or SMT+PE.** **A-C**. 28-day (A), 90-day (B), and 1-year (C) survival curves for HBV-ACLF patients with MELD grade 2. **D-F**. 28-day (D), 90-day (E), and 1-year (F) survival curves for HBV-ACLF patients with MELD grade 4. **G-I**. 28-day (G), 90-day (H), and 1-year (I) survival curves for HBV-ACLF patients with ACLF grade 3. SMT, standard medical therapy; PE, plasma exchange; HBV-ACLF, hepatitis B virus-associated acute-on-chronic liver failure.

**Fig. S3. Changes in SIRS scores in166 pairs of matched patients with HBV-ACLF who received SMT alone or SMT+PE.** SIRS, systemic inflammatory response syndrome; SMT, standard medical therapy; PE, plasma exchange; HBV-ACLF, hepatitis B virus-associated acute-on-chronic liver failure.

**Supplementary table 1 Prognostic factors at 28 days, 90 days, and 1 year in HBV-ACLF patients treated with SMT alone or in combination with SMT and PE-based NB-ALSS (Univariate COX regression analysis)**

| **Univariate** | p value | HR | 95% CI HR | | Time point |
| --- | --- | --- | --- | --- | --- |
| Parameter |  |  | Lower Bound | Upper Bound |  |
| PE (yes) | 0.007 | 0.601 | 0.414 | 0.871 | 28 day |
| Age (year) | <0.001 | 1.042 | 1.025 | 1.059 |  |
| Sex (male) | 0.610 | 0.865 | 0.494 | 1.513 |  |
| HE (≥3) (yes) | <0.001 | 3.203 | 1.910 | 5.374 |  |
| Respiratory failure (yes) | 0.003 | 2.452 | 1.347 | 4.463 |  |
| Circulatory failure (yes) | 0.332 | 1.999 | 0.494 | 8.095 |  |
| Infection (yes) | 0.103 | 1.368 | 0.939 | 1.994 |  |
| Meld score | <0.001 | 1.072 | 1.040 | 1.104 |  |
| Meld-Na score | <0.001 | 1.085 | 1.050 | 1.121 |  |
| CRP (μg/L) | 0.009 | 1.013 | 1.003 | 1.023 |  |
| ALT (U/L) | 0.696 | 1.000 | 1.000 | 1.000 |  |
| AST (U/L) | 0.777 | 1.000 | 1.000 | 1.000 |  |
| GGT (U/L) | 0.450 | 1.001 | 0.998 | 1.004 |  |
| AKP (U/L) | 0.041 | 0.996 | 0.993 | 1.000 |  |
| Tbil (μmol/L) | 0.030 | 1.001 | 1.000 | 1.002 |  |
| Alb (g/L) | 0.836 | 0.996 | 0.961 | 1.033 |  |
| Na^+^ (mmol/L) | 0.018 | 0.960 | 0.929 | 0.993 |  |
| K^+^ (mmol/L) | 0.488 | 0.904 | 0.680 | 1.202 |  |
| Cr (μmol/L) | 0.279 | 1.001 | 0.999 | 1.003 |  |
| BUN (mmol/L) | 0.572 | 1.003 | 0.992 | 1.014 |  |
| PT (s) | 0.052 | 1.013 | 1.000 | 1.027 |  |
| INR | <0.001 | 1.233 | 1.113 | 1.365 |  |
| WBC (×10^9^/L) | 0.117 | 1.035 | 0.991 | 1.082 |  |
| HGB (g/L) | 0.830 | 0.999 | 0.991 | 1.007 |  |
| PLT (×10^9^/L) | 0.129 | 0.997 | 0.993 | 1.001 |  |
| PE (yes) | 0.033 | 0.720 | 0.532 | 0.974 | 90 day |
| Age (year) | <0.001 | 1.040 | 1.026 | 1.053 |  |
| Sex (male) | 0.775 | 0.938 | 0.604 | 1.456 |  |
| HE (≥3) (yes) | <0.001 | 2.652 | 1.642 | 4.284 |  |
| Respiratory failure (yes) | 0.006 | 2.147 | 1.240 | 3.714 |  |
| Circulatory failure (yes) | 0.584 | 1.476 | 0.366 | 5.954 |  |
| Infection (yes) | 0.230 | 1.211 | 0.886 | 1.655 |  |
| Meld score | <0.001 | 1.072 | 1.044 | 1.101 |  |
| Meld-Na score | <0.001 | 1.087 | 1.057 | 1.118 |  |
| CRP (μg/L) | 0.021 | 1.011 | 1.002 | 1.021 |  |
| ALT (U/L) | 0.986 | 1.000 | 1.000 | 1.000 |  |
| AST (U/L) | 0.718 | 1.000 | 1.000 | 1.000 |  |
| GGT (U/L) | 0.151 | 1.002 | 0.999 | 1.004 |  |
| AKP (U/L) | 0.047 | 0.997 | 0.995 | 1.000 |  |
| Tbil (μmol/L) | <0.001 | 1.002 | 1.001 | 1.003 |  |
| Alb (g/L) | 0.877 | 0.998 | 0.969 | 1.027 |  |
| Na^+^ (mmol/L) | 0.001 | 0.953 | 0.927 | 0.980 |  |
| K^+^ (mmol/L) | 0.495 | 0.923 | 0.733 | 1.162 |  |
| Cr (μmol/L) | 0.210 | 1.001 | 0.999 | 1.003 |  |
| BUN (mmol/L) | 0.595 | 1.002 | 0.993 | 1.012 |  |
| PT (s) | 0.005 | 1.016 | 1.005 | 1.028 |  |
| INR | <0.001 | 1.246 | 1.138 | 1.365 |  |
| WBC (×10^9^/L) | 0.081 | 1.033 | 0.996 | 1.071 |  |
| HGB (g/L) | 0.515 | 0.998 | 0.991 | 1.004 |  |
| PLT (×10^9^/L) | 0.015 | 0.996 | 0.993 | 0.999 |  |
| PE (yes) | 0.016 | 0.701 | 0.526 | 0.935 | 1 year |
| Age (year) | <0.001 | 1.036 | 1.023 | 1.049 |  |
| Sex (male) | 0.740 | 0.931 | 0.611 | 1.419 |  |
| HE (≥3) (yes) | <0.001 | 2.411 | 1.497 | 3.883 |  |
| Respiratory failure (yes) | 0.014 | 1.981 | 1.147 | 3.421 |  |
| Circulatory failure (yes) | 0.721 | 1.290 | 0.320 | 5.197 |  |
| Infection (yes) | 0.721 | 1.290 | 0.320 | 5.197 |  |
| Meld score | <0.001 | 1.070 | 1.043 | 1.098 |  |
| Meld-Na score | <0.001 | 1.085 | 1.055 | 1.115 |  |
| CRP (μg/L) | 0.024 | 1.011 | 1.001 | 1.020 |  |
| ALT (U/L) | 0.786 | 1.000 | 1.000 | 1.000 |  |
| AST (U/L) | 0.658 | 1.000 | 1.000 | 1.000 |  |
| GGT (U/L) | 0.151 | 1.002 | 0.999 | 1.004 |  |
| AKP (U/L) | 0.074 | 0.998 | 0.995 | 1.000 |  |
| Tbil (μmol/L) | <0.001 | 1.002 | 1.001 | 1.003 |  |
| Alb (g/L) | 0.862 | 0.998 | 0.970 | 1.026 |  |
| Na^+^ (mmol/L) | <0.001 | 0.952 | 0.927 | 0.979 |  |
| K^+^ (mmol/L) | 0.472 | 0.923 | 0.743 | 1.148 |  |
| Cr (μmol/L) | 0.277 | 1.001 | 0.999 | 1.002 |  |
| BUN (mmol/L) | 0.719 | 1.002 | 0.993 | 1.011 |  |
| PT (s) | 0.001 | 1.018 | 1.007 | 1.028 |  |
| INR | <0.001 | 1.240 | 1.133 | 1.358 |  |
| WBC (×10^9^/L) | 0.044 | 1.036 | 1.001 | 1.072 |  |
| HGB (g/L) | 0.617 | 0.998 | 0.992 | 1.005 |  |
| PLT (×10^9^/L) | 0.012 | 0.996 | 0.993 | 0.999 |  |

**Abbreviations**: HBV-ACLF, hepatitis B virus-associated acute-on-chronic liver failure; PE, plasma exchange; HE, hepatic encephalopathy; Meld score, Model for end-stage liver disease score; Na^+^, serum sodium; INR, international normalized ratio; CRP, c-reactive protein; HR, hazard ratio.

**Supplementary table 2 Prognostic factors in HBV-ACLF patients treated with PE-based NB-ALSS (Univariate COX regression analysis)**

| **Univariate** | p value | HR | 95% CI HR | | Time point |
| --- | --- | --- | --- | --- | --- |
| Parameter |  |  | Lower Bound | Upper Bound |  |
| PE times | 0.010 | 0.723 | 0.565 | 0.926 | 28 day |
| Mean plasma dose | 0.508 | 1.000 | 0.999 | 1.000 |  |
| Therapy timing | 0.075 | 0.947 | 0.891 | 1.006 |  |
| Age (year) | 0.001 | 1.042 | 1.016 | 1.068 |  |
| Sex (male) | 0.782 | 0.887 | 0.378 | 2.081 |  |
| HE (≥3) (yes) | 0.043 | 2.596 | 1.029 | 6.546 |  |
| Respiratory failure (yes) | 0.042 | 2.427 | 1.033 | 5.702 |  |
| Circulatory failure (yes) | 0.694 | 1.489 | 0.206 | 10.788 |  |
| Infection (yes) | 0.402 | 0.768 | 0.414 | 1.424 |  |
| Meld score | 0.004 | 1.065 | 1.021 | 1.112 |  |
| Meld-Na score | 0.002 | 1.075 | 1.026 | 1.127 |  |
| CRP (μg/L) | <0.001 | 1.020 | 1.009 | 1.031 |  |
| ALT (U/L) | 0.017 | 1.001 | 1.000 | 1.001 |  |
| AST (U/L) | 0.154 | 1.000 | 1.000 | 1.001 |  |
| GGT (U/L) | 0.154 | 1.002 | 0.999 | 1.006 |  |
| AKP (U/L) | 0.080 | 0.996 | 0.991 | 1.001 |  |
| Tbil (μmol/L) | 0.690 | 1.000 | 0.999 | 1.002 |  |
| Alb (g/L) | 0.615 | 0.986 | 0.933 | 1.042 |  |
| Na^+^ (mmol/L) | 0.128 | 0.964 | 0.920 | 1.010 |  |
| K^+^ (mmol/L) | 0.509 | 1.155 | 0.753 | 1.771 |  |
| Cr (μmol/L) | 0.111 | 1.003 | 0.999 | 1.007 |  |
| BUN (mmol/L) | 0.001 | 1.057 | 1.022 | 1.092 |  |
| PT (s) | 0.108 | 1.017 | 0.996 | 1.037 |  |
| INR | <0.001 | 1.295 | 1.124 | 1.492 |  |
| WBC (×10^9^/L) | 0.500 | 1.022 | 0.960 | 1.087 |  |
| HGB (g/L) | 0.368 | 1.006 | 0.993 | 1.019 |  |
| PLT (×10^9^/L) | 0.822 | 1.001 | 0.994 | 1.007 |  |
| PE times | 0.008 | 0.781 | 0.651 | 0.937 | 90 day |
| Mean plasma dose | 0.242 | 1.000 | 1.000 | 1.000 |  |
| Therapy timing | 0.182 | 0.974 | 0.937 | 1.012 |  |
| Age (year) | <0.001 | 1.042 | 1.022 | 1.063 |  |
| Sex (male) | 0.957 | 0.983 | 0.521 | 1.855 |  |
| HE (≥3) (yes) | 0.075 | 2.128 | 0.925 | 4.894 |  |
| Respiratory failure (yes) | 0.021 | 2.367 | 1.137 | 4.926 |  |
| Circulatory failure (yes) | 0.977 | 0.971 | 0.135 | 6.981 |  |
| Infection (yes) | 0.338 | 0.795 | 0.496 | 1.272 |  |
| Meld score | 0.010 | 1.052 | 1.012 | 1.094 |  |
| Meld-Na score | 0.004 | 1.063 | 1.020 | 1.109 |  |
| CRP (μg/L) | 0.001 | 1.019 | 1.007 | 1.030 |  |
| ALT (U/L) | 0.055 | 1.000 | 1.000 | 1.001 |  |
| AST (U/L) | 0.129 | 1.000 | 1.000 | 1.001 |  |
| GGT (U/L) | 0.014 | 1.003 | 1.001 | 1.006 |  |
| AKP (U/L) | 0.137 | 0.997 | 0.994 | 1.001 |  |
| Tbil (μmol/L) | 0.118 | 1.001 | 1.000 | 1.002 |  |
| Alb (g/L) | 0.649 | 0.990 | 0.949 | 1.033 |  |
| Na^+^ (mmol/L) | 0.036 | 0.960 | 0.925 | 0.997 |  |
| K^+^ (mmol/L) | 0.707 | 1.067 | 0.760 | 1.498 |  |
| Cr (μmol/L) | 0.362 | 1.002 | 0.998 | 1.005 |  |
| BUN (mmol/L) | 0.001 | 1.054 | 1.023 | 1.087 |  |
| PT (s) | 0.009 | 1.022 | 1.006 | 1.039 |  |
| INR | <0.001 | 1.277 | 1.117 | 1.460 |  |
| WBC (×10^9^/L) | 0.260 | 1.028 | 0.980 | 1.079 |  |
| HGB (g/L) | 0.906 | 1.001 | 0.990 | 1.011 |  |
| PLT (×10^9^/L) | 0.511 | 0.998 | 0.993 | 1.003 |  |
| PE times | 0.025 | 0.827 | 0.700 | 0.977 | 1 year |
| Mean plasma dose | 0.640 | 1.000 | 1.000 | 1.000 |  |
| Therapy timing | 0.625 | 0.992 | 0.961 | 1.024 |  |
| Age (year) | <0.001 | 1.038 | 1.019 | 1.057 |  |
| Sex (male) | 0.722 | 0.892 | 0.475 | 1.676 |  |
| HE (≥3) (yes) | 0.121 | 1.928 | 0.841 | 4.420 |  |
| Respiratory failure (yes) | 0.035 | 2.188 | 1.055 | 4.537 |  |
| Circulatory failure (yes) | 0.874 | 0.853 | 0.119 | 6.125 |  |
| Infection (yes) | 0.413 | 0.831 | 0.533 | 1.295 |  |
| Meld score | 0.024 | 1.046 | 1.006 | 1.087 |  |
| Meld-Na score | 0.008 | 1.057 | 1.015 | 1.102 |  |
| CRP (μg/L) | 0.001 | 1.018 | 1.007 | 1.030 |  |
| ALT (U/L) | 0.076 | 1.000 | 1.000 | 1.001 |  |
| AST (U/L) | 0.115 | 1.000 | 1.000 | 1.001 |  |
| GGT (U/L) | 0.012 | 1.003 | 1.001 | 1.006 |  |
| AKP (U/L) | 0.209 | 0.998 | 0.995 | 1.001 |  |
| Tbil (μmol/L) | 0.089 | 1.001 | 1.000 | 1.002 |  |
| Alb (g/L) | 0.838 | 0.996 | 0.958 | 1.036 |  |
| Na^+^ (mmol/L) | 0.036 | 0.961 | 0.927 | 0.997 |  |
| K^+^ (mmol/L) | 0.710 | 1.063 | 0.770 | 1.468 |  |
| Cr (μmol/L) | 0.387 | 1.001 | 0.998 | 1.005 |  |
| BUN (mmol/L) | 0.001 | 1.053 | 1.022 | 1.084 |  |
| PT (s) | 0.005 | 1.023 | 1.007 | 1.039 |  |
| INR | 0.001 | 1.257 | 1.096 | 1.442 |  |
| WBC (×10^9^/L) | 0.217 | 1.030 | 0.983 | 1.078 |  |
| HGB (g/L) | 0.801 | 1.001 | 0.992 | 1.011 |  |
| PLT (×10^9^/L) | 0.517 | 0.998 | 0.994 | 1.003 |  |

**Abbreviations**: PE, plasma exchange; HE, hepatic encephalopathy; INR, international normalized ratio; BUN, blood urea nitrogen; CRP, c-reactive protein; PT, prothrombin time; HR, hazard ratio.
